# Alcohol use disorder and its association with personal well-being and life satisfaction

**DOI:** 10.1192/bjo.2021.709

**Published:** 2021-06-18

**Authors:** Chau Sian Lim, Zhen Wei Lew, Yoke Chiang Ng, Lai Huat Peh, Hatta Santoso Ong

**Affiliations:** Changi General Hospital

## Abstract

**Aims:**

This study aims to find out how alcohol use disorder (AUD) correlates to personal well-being and life satisfaction.

**Background:**

AUD is prevalent and leads to significant physical, physiological, and social-occupational impairment. Mental well-being involves the overall positive psychological state of a person – being well adjusted, socially engaged, and emotionally healthy. Despite the paradigm shift from purely treating mental illness to promoting positive mental health, there is limited literature describing the relationship between alcohol use disorder and mental well-being.

**Method:**

This cross-sectional study was conducted in a general hospital in Singapore. Patients admitted across a span of two years were screened for possible alcohol use disorder. Patients were included if they were male, aged 21 years and above, and had the mental capacity to give consent. They were excluded if they had illicit drug use, acute mental illness, inability or refusal to give consent, or if they were already receiving intervention for addiction issues. Participants were administered the Alcohol Use Disorders Identification Test (AUDIT). Those who scored 8 or above were classified as being at risk for AUD, while those who scored 7 or less were classified as at low risk. They were also administered the Personal Wellbeing Index (PWI) and the “Satisfaction with Life as a Whole” question. The PWI measures individuals’ subjective well-being across seven domains. The “Satisfaction with Life as a Whole” question measures, on an eleven-point Likert scale, how satisfied the respondent feels with life in general. Demographic data were also collected and STATA v. 12.1 was used for statistical analysis.

**Result:**

Among a total of 134 participants, 25 of them scored ≥8 on the AUDIT and 109 scored 7 or less. On the PWI, the group at risk scored significantly lower at 71.3 (95% CI: 66.0–76.7) compared to the group not at risk at 77.9 (95% CI: 75.8–79.9), p < 0.01. The results were similar on the “Satisfaction with Life as a Whole” item. The group at risk had a mean of 6.72 (95% CI: 6.03–7.41) while the group not at risk had a mean of 7.67 (95% CI: 7.41–7.93), both p < 0.01. The differences between the high risk and low risk groups remained statistically significant even after adjusting for differences in age, race, education level, and employment status.

**Conclusion:**

This study demonstrated a statistically significant association between AUD and personal well-being as well as satisfaction with life among males.

